# The silent waitress. A case report of mutism without catatonia

**DOI:** 10.1192/j.eurpsy.2023.1306

**Published:** 2023-07-19

**Authors:** T. Fernández, E. Pujal, S. Salmeron, M. Bioque

**Affiliations:** Psychiatry, Hospital Clinic Barcelona, Barcelona, Spain

## Abstract

**Introduction:**

Mutism, defined as an inability or unwillingness to speak, resulting in an absence or reduction of speech, has a wide differential diagnosis. It rarely presents as an isolated disability and often occurs in association with other disturbances in behavior, thought processes, affect, or level of consciousness. Mutism is typically associated with catatonia, usually in schizophrenia, but also depression, bipolar disorder, intoxication, and neurological conditions.

**Objectives:**

To describe a case of mutism without catatonia.

**Methods:**

We describe a clinical case of a patient admitted to our psychiatric inpatient unit with mutism as the presenting symptom. The literature on this subject is also selectively reviewed.

**Results:**

A 49-year-old woman was found mute at home by her brother and brought to our emergency room. Not a word had come out of his mouth for the past month. She would show up at the restaurant where she worked as a waitress and do her job, but she didn’t talk. As a result, she had been fired. Her routine daily chores and her vegetative functions were maintained. She had no prior history of medical or psychiatric illness or substance abuse. In addition to the mutism, the patient showed an important psychomotor restlessness and performed repetitive hand movements suggestive of occupational delirium. There was no rigidity, stupor, negativism, catalepsy, echosymptoms or any other catatonic symptomatology.

She was then admitted to our inpatient unit, where a complete blood test, EKG, brain CT, brain MRI, EEG and a lumbar puncture with biochemistry and neuroimmunology studies were performed, none of them showing any abnormalities.

The clinical presentation suggested the diagnosis of either a psychotic disorder or a major depressive episode.

The patient was then started on olanzapine up to 20 mg/d, fluoxetine up to 20 mg/d and lorazepam up to 6 mg/d. Due to persistence of symptomatology despite pharmacological treatment, she was started on Electroconvulsive Therapy (ECT). At the time of issuance of this report, 7 bilateral ECT courses have been carried out and absolute mutism persists. Although she has presented an improvement of the anxiety and the repetitive behaviors noted on admission have disappeared, she hasn´t resumed speaking.

**Conclusions:**

Mutism occurs in a number of conditions, both functional and organic, and an accurated diagnosis is important for the management. One must perform a thorough physical and systemic examination to rule out organic causes for mutism. An observation for some time period may be warranted and should be done to reach final diagnosis in our case.

**Disclosure of Interest:**

None Declared

